# Platelet Turnover in Stable Coronary Artery Disease – Influence of Thrombopoietin and Low-Grade Inflammation

**DOI:** 10.1371/journal.pone.0085566

**Published:** 2014-01-21

**Authors:** Sanne Bøjet Larsen, Erik Lerkevang Grove, Anne-Mette Hvas, Steen Dalby Kristensen

**Affiliations:** 1 Department of Cardiology, Aarhus University Hospital, Aarhus, Denmark; 2 Department of Clinical Biochemistry, Aarhus University Hospital, Aarhus, Denmark; 3 Faculty of Health Sciences, Aarhus University, Aarhus, Denmark; King’s College London School of Medicine, United Kingdom

## Abstract

**Background:**

Newly formed platelets are associated with increased aggregation and adverse outcomes in patients with coronary artery disease (CAD). The mechanisms involved in the regulation of platelet turnover in patients with CAD are largely unknown.

**Aim:**

To investigate associations between platelet turnover parameters, thrombopoietin and markers of low-grade inflammation in patients with stable CAD. Furthermore, to explore the relationship between platelet turnover parameters and type 2 diabetes, prior myocardial infarction, smoking, age, gender and renal insufficiency.

**Methods:**

We studied 581 stable CAD patients. Platelet turnover parameters (immature platelet fraction, immature platelet count, mean platelet volume, platelet distribution width and platelet large cell-ratio) were determined using automated flow cytometry (Sysmex XE-2100). Furthermore, we measured thrombopoietin and evaluated low-grade inflammation by measurement of high-sensitive CRP and interleukin-6.

**Results:**

We found strong associations between the immature platelet fraction, immature platelet count, mean platelet volume, platelet distribution width and platelet large cell ratio (r = 0.61–0.99, p<0.0001). Thrombopoietin levels were inversely related to all of the platelet turnover parameters (r = −0.17–−0.25, p<0.0001). Moreover, thrombopoietin levels were significantly increased in patients with diabetes (p = 0.03) and in smokers (p = 0.003). Low-grade inflammation evaluated by high-sensitive CRP correlated significantly, yet weakly, with immature platelet count (r = 0.10, p = 0.03) and thrombopoietin (r = 0.16, p<0.001). Also interleukin-6 correlated with thrombopoietin (r = 0.10, p = 0.02).

**Conclusion:**

In stable CAD patients, thrombopoietin was inversely associated with platelet turnover parameters. Furthermore, thrombopoietin levels were increased in patients with diabetes and in smokers. However, low-grade inflammation did not seem to have a substantial impact on platelet turnover parameters.

## Introduction

Platelets are key players in the development of coronary atherothrombosis, which is the main cause of acute coronary syndromes. Within an individual, platelets are heterogeneous in both size and density. The circulating pool of platelets is held in an equilibrium, which is balanced by platelet production and consumption. In patients with increased platelet turnover, a larger population of young platelets in peripheral blood can now be identified and quantified by staining for messenger ribonucleic acid (mRNA) using either manual techniques [1] or automated flow cytometry [2]. These newly formed platelets, often referred to as “reticulated” or “immature” platelets, lack genomic DNA but contain megakaryocyte-derived mRNA and thus have the translational capacity necessary for protein synthesis [3]. Moreover, immature platelets are characterized by a higher number of dense granules and an increased platelet volume than older platelets [1]. Finally, larger platelets have been shown to be enzymatically and metabolically more active and to have a higher thrombotic potential than smaller platelets [4]–[6].

Mean platelet volume (MPV) has been used as a surrogate marker of platelet turnover and has been shown to be increased in the acute phase of myocardial infarction [7] and also to be a predictor of adverse cardiovascular outcomes in healthy subjects [8] and in patients with previous myocardial infarction [9]. Furthermore, MPV has been reported to be increased in patients with cardiovascular risk factors such as diabetes mellitus [10], smoking [11] and obesity [12].

Increased platelet consumption has been described in patients with coronary atherosclerosis and may be explained by a pathophysiological interaction between platelets and atherosclerotic vessels [13]. Several studies have reported increased levels of immature platelets in patients with acute coronary syndrome [7], [14]–[17] and in patients with previous stent thrombosis [18], [19]. Moreover, high levels of immature platelets are associated with increased residual platelet aggregation in stable patients with coronary artery disease (CAD) receiving antiplatelet therapy [18], [20], [21]. Finally, immature platelets have been shown to be independent predictors of cardiovascular death in patients with acute coronary syndrome [22].

Some studies have investigated platelet volume indices in stable CAD patients [10], [16], [17], [20]. Only a previous study from our group [20] has included the multitude of platelet turnover parameters as evaluated in the present study. Still, gaps of knowledge exist regarding platelet turnover parameters in stable CAD patients. Thrombopoietin and interleukin-6 (IL-6) have been suggested as important regulators of platelet production, yet the mechanisms involved in platelet production and increased turnover are largely unknown [7], [23]. Furthermore, only sparse data exists about the impact of thrombopoietin and low-grade inflammation on platelet turnover in stable CAD patients.

In this hypothesis-generating study, we investigated associations between platelet turnover parameters, thrombopoietin and markers of low-grade inflammation in stable, high-risk CAD patients receiving low-dose aspirin as mono antiplatelet therapy. Furthermore, we explored if platelet turnover parameters were related to the presence of type 2 diabetes, prior myocardial infarction, current smoking, age, gender or renal insufficiency.

## Methods

### Study Population

We performed a cross-sectional study including 581 stable patients with angiographically documented CAD. In addition, all patients had either prior myocardial infarction (at least 12 months ago), type 2 diabetes mellitus or both. Patients were recruited from the Western Denmark Heart Registry [24] and enrolled from February 2009 to January 2011.

All diabetic patients were diagnosed with type 2 diabetes and treated with oral antidiabetic drugs and/or insulin. All non-diabetic patients had fasting plasma glucose levels <7.0 mmol/L at the time of inclusion. Smokers were defined as active smokers at the time of blood sampling and non-smokers consisted of never smokers and previous smokers. All patients included in the study were treated with 75 mg non-enteric coated aspirin once daily as mono antiplatelet therapy. Patients with platelet counts <120×10^9^/L or >450×10^9^/L were excluded. The in- and exclusion criteria have previously been described in detail [25].

### Ethics Statement

The study was conducted in agreement with the Helsinki-II-declaration and approved by The Central Denmark Region Committees on Health Research (M-2007-0180, M-2009-0110) and by the Danish Data Protection Agency. All patients gave written informed consent.

### Laboratory Investigations

#### Blood sampling

All samples were obtained from the antecubital vein with patients in supine position after 30 minutes of rest using vacuum tubes, a large bore needle (19 G), and a minimum of stasis.

#### Platelet characteristics and haematological parameters

Blood samples for haematological analyses were collected in 3.0 mL tubes containing EDTA (Terumo, Leuven, Belgium). In order to minimize and standardize time-dependent swelling of platelets, haematological analyses were performed within 60 minutes of blood sampling. Haematology parameters were measured using the Sysmex XE-2100 hematology analyser (Sysmex, Kobe, Japan) with upgraded software (XE IPF Master, Sysmex) allowing flow cytometric detection of immature platelets as previously described [2], [20]. In brief, platelet RNA was stained with flourescent dyes (polymethine and oxazine) before stained cells were passed through a semiconductor diode laser beam. The resulting flourescence intensity (RNA content) and forward light scatter (cell volume) were measured and a preset gate (XE IPF Master software, Sysmex) discriminated between mature and immature platelets. Absolute immature platelet count (IPC) was obtained, and immature platelet fraction (IPF) was calculated as the ratio of immature platelets to the total platelet count and reported in percent. Platelet volume parameters were derived from the platelet volume distribution. MPV was calculated by dividing the platelet crit by platelet impedance count. Platelet distribution width (PDW), a measure of platelet anisocytosis, was the width of the size distribution curve in femtoliters (fL) at the 20% level of the peak. The platelet large cell ratio (P-LCR) was defined as the number of cells falling above the 12-fL threshold divided by platelet count.

#### Thrombopoietin

Whole blood was allowed to clot at room temperature for 30 minutes before serum was separated by centrifugation at 1000 g for 15 minutes and stored at −80°C until analysis. Thrombopoietin concentrations were analysed in duplicate for all patients and the mean of the two results included in the statistical analyses. The coefficient of variance was 9%. Thrombopoietin analyses were performed by ELISA according to the manufacturer’s instructions (Human Tpo Immunoassay, R&D Systems Europe Ltd., Abingdon, UK)

#### Inflammatory markers

Blood for IL-6 analyses (cobas® 6000 analyser, E module, Roche, Mannheim, Germany) was collected in non-siliconized 5.0 mL tubes (Terumo, Leuven, Belgium) without anticoagulants. The blood was allowed to clot for one hour at 37°C before serum was separated by centrifugation at 2600 g for 10 minutes. Serum was stored at −80°C until analysis.

Blood for high-sensitive C-reactive protein (hs-CRP) analyses (KoneLab 30i, ILS Laboratories Scandinavia, Allerød, Denmark) was collected in 3.0 mL lithium-heparin tubes containing separating gel (Terumo, Leuven, Belgium). The measurement interval for hs-CRP was 0.2–10.0 mg/L. A total of 493 (85%) of the 581 patients were included in the hs-CRP analyses; 28 patients had hs-CRP levels >10.0 mg/L, and hs-CRP was not measured in 57 patients due to a change in laboratory procedures regarding measurements of hs-CRP during the study.

### Statistics

If normally distributed, continuous data is presented as mean and standard deviation (SD), if not as median and interquartile range (IQR). Differences between two unpaired groups were tested with a two-sided t-test if data was normally distributed; otherwise the Mann-Whitney test was used. Proportions between two groups were tested using Fischer’s exact test and presented as absolute counts and percentages. Correlations were performed using Spearman’s rank coefficient. Multiple linear regression analyses were used to identify independent determinants of platelet turnover parameters. In the regression analyses, observations which were missing on the outcome variable or on any of the predictor variables were removed. A two-sided p-value <0.05 was considered statistically significant. Data was registered in Epidata® version 3.1 (EpiData Association, Odense Denmark). Statistical analyses were performed using Stata® version 11.0 (StataCorp LP, TX, USA) and graphs performed using GraphPad Prism® version 5.0 (GraphPad Software, CA, USA).

## Results

### Study Population

Clinical and biochemical characteristics of the study population are shown in Table 1. We studied a population of stable CAD patients with a relatively high-risk profile, as 92% of the patients had a history of prior myocardial infarction, 25% had type 2 diabetes and 17% had both.

**Table 1:** Baseline characteristics of the study population, n = 581.

Age, years	64±9
Body mass index, kg/m2	28±4
Males	460 (79)
Current smokers	122 (21)
Blood pressure, systolic, mm Hg	142±20
Blood pressure, diastolic, mm Hg	82±11
*Biochemistry*	
B-Leukocytes, 10^9^/L	7.0±1.9
B-Haemoglobin, mmol/L	8.9±0.7
B-Red blood cell count, 10^12^/L	4.8±0.4
B-Reticulocyte count, 10^9^/L	48±15
B-Platelet count, 10^9^/L	231±59
B-Immature platelet count, 10^9^/L	5.8 (4.4;7.9)
B-Immature platelet fraction, %	2.5 (1.9;3.5)
B-Mean platelet volume, fL	10.8±0.9
B-Platelet distribution width, fL	13.4±1.9
B-Platelet large cell ratio, %	32±7
P-High-sensitive C-reactive protein, mg/L	0.8 (0.4;1.6)
S-Interleukin-6, pg/mL	2.2 (1.5;3.4)
S-Thrombopoietin, pg/mL	45 (28;64)
P-Creatinine, µmol/L	81 (71;94)
*Cardiovascular morbidity*	
Prior percutaneus coronary intervention	562 (97)
Prior myocardial infarction	533 (92)
Prior coronary artey bypass grafting	49 (8)
Prior stroke	25(4)
Type 2 Diabetes Mellitus	148 (25)
*Medication*	
Aspirin	581 (100)
Statins	533 (92)
Beta-blockers	440 (76)
ACE inhibitors	265 (46)
Angiotensin receptor blockers	80 (14)
Calcium antagonists	111 (19)
Diuretics	147 (25)
Proton pump inhibitors	65 (11)
Insulin^a^	47 (32)
Oral antidiabetic medication^a^	129 (87)

Data is presented as mean±SD, n(%) or median (25th;75th percentile).

^a^ out of 148 patients with type 2 diabetes.

B: blood, S: serum, P: plasma.

### Platelet Turnover Parameters

Correlation analyses between platelet parameters are listed in Table 2. As expected, all platelet turnover parameters were moderately or strongly associated. A total of 35 patients had high IPF values (≥6%, range 6–14%). These patients were older (68±1.2 vs. 64±0.4 years, p = 0.02) and more frequently had a history of prior myocardial infarction (83% vs. 17%, p = 0.05).

**Table 2:** Correlation analyses between platelet parameters, n = 581.

	IPF, %	IPC, 10^9^/L	MPV, fL	PC, 10^9^/L	PDW, fL
**IPF, %**	/	/	/	/	/
					
**IPC, 10^9^/L**	r = 0.84	/	/	/	/
	p<0.0001				
**MPV, fL**	r = 0.78	r = 0.64	/	/	/
	p<0.0001	p<0.0001			
**PC, 10^9^/L**	r = −0.41	r = 0.11	r = 0.36	/	/
	p<0.0001	p = 0.0070	p<0.0001		
**PDW, fL**	r = 0.76	r = 0.61	r = 0.94	r = −0.37	/
	p<0.0001	p<0.0001	p<0.0001	p<0.0001	
**P-LCR, %**	r = 0.78	r = 0.63	r = 0.99	r = −0.37	r = 0.96
	p<0.0001	p<0.0001	p<0.0001	p<0.0001	p<0.0001

IPF: Immature platelet fraction, IPC: Immature platelet count, MPV: Mean platelet volume.

PC: Platelet count, PDW: Platelet distribution width, P-LCR: Platelet large cell ratio.

### Platelet Turnover Parameters, Thrombopoietin and Low-grade Inflammation

**Figure 1:**
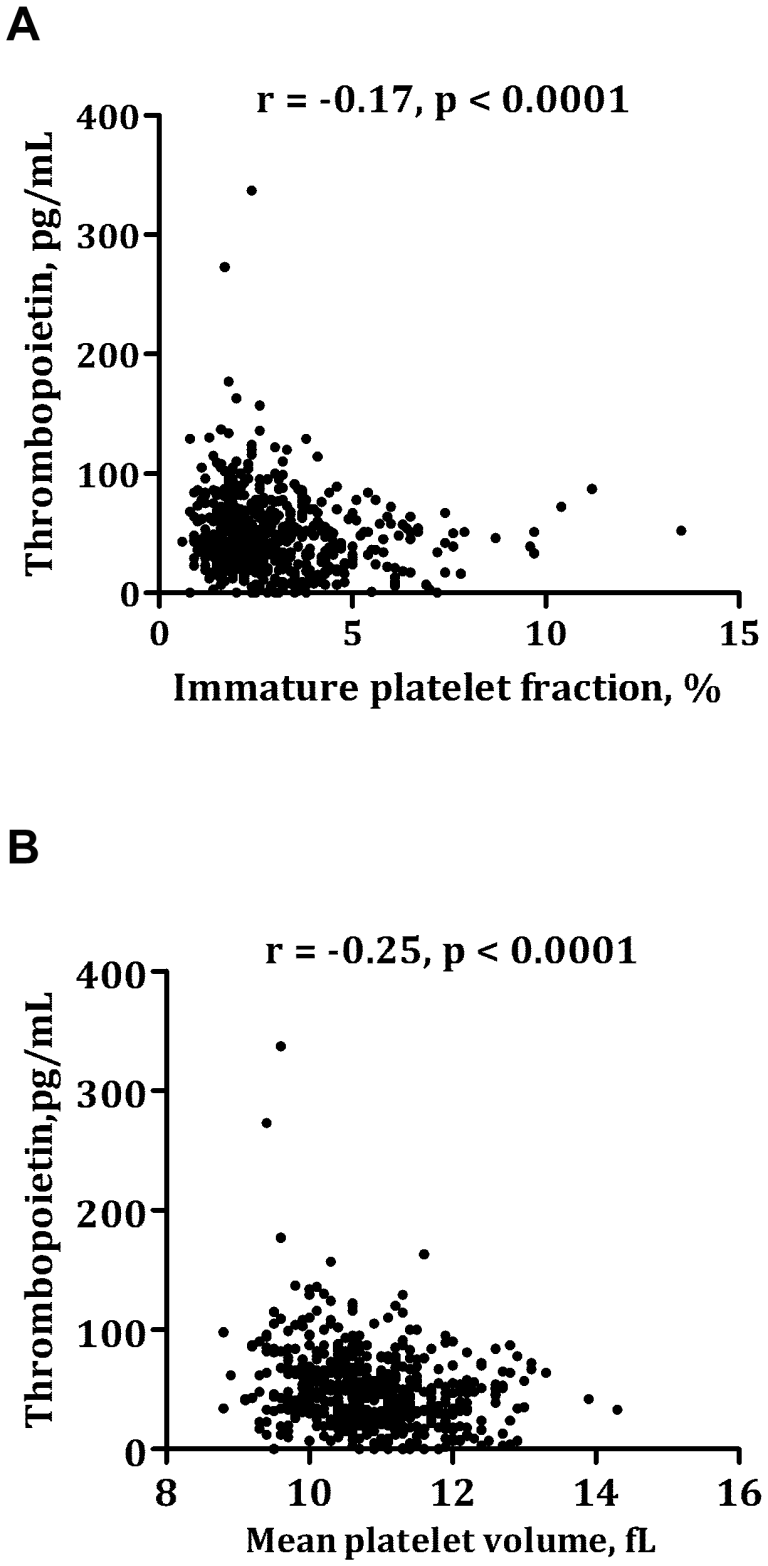
Correlation between thrombopoietin and immature platelet fraction (Figure 1A) and mean platelet volume (Figure 1B).

Thrombopoietin was inversely related to IPF (Figure 1A), MPV (Figure 1B), IPC, PDW and P-LCR (r = −0.17–−0.25, p<0.0001). There was a significant, yet weak, association between thrombopoietin and hs-CRP (r = 0.10, p = 0.03) and IL-6 (r = 0.10, p = 0.02). No association was found between thrombopoietin and platelet count (r = −0.01, p = 0.86). Although platelet counts were in the normal range (between 120 and 450^∧^10^9^/L), five patients had very high thrombopoietin levels (>150 pg/mL) (Figure 1). Both hs-CRP and IL-6 were numerically elevated in these five patients as compared with patients having thrombopoietin levels <150 pg/mL (median hs-CRP mg/L [IQR]: 2.1 [0.7;2,5] vs. 0.9 [0.5;2.2], median IL-6 pg/mL [IQR]: 2.3 [1.7;6.6] vs. 2.2 [1.5;3.4]).

Hs-CRP was significantly, however weakly, correlated with IPC (r = 0.10, p = 0.03) and platelet count (r = 0.11, p = 0.01). IL-6 did not correlate with any of the platelet turnover parameters (data not shown).

### Platelet Turnover Parameters and Clinical Characteristics

Platelet parameters and clinical characteristics of the study population are summarized in Table 3. Patients with diabetes had significantly increased PDW and slightly augmented IPC, MPV and P-LCR, however, this did not reach statistical significance. Smokers had significantly higher IPC and platelet counts than non-smokers. Females, patients aged ≥65 years and patients with an estimated glomerular filtration rate (eGFR) ≤60 mL/min had significantly increased platelet counts. Furthermore, females had higher IPF than males. Platelet turnover parameters did not differ between patients with or without prior myocardial infarction. Except for age and platelet count, all of the above-mentioned differences remained statistically significant after adjustment for diabetes, prior myocardial infarction, smoking, sex, age and eGFR in linear multivariate regression analyses (Table 3).

**Table 3:** Platelet parameters and clinical characteristics, n = 581.

	Immature		Immature		Mean platelet	
	platelet fraction, %		platelet count, 10^∧^9/L		volume, fL	
**No diabetes**	2.5 (1.9;3.5)	p = 0.15	5.7 (4.3;7.6)	p = 0.07§	10.7 (10.1;11.3)	p = 0.06§
**Type 2 diabetes**	2.6 (2.0;3.7)		6.1 (4.5;8.4)		10.9 (10.3;11.5)	
**No prior MI**	2.4 (1.8;3.7)	p = 0.99	5.4 (4.5;7.8)	p = 0.76	10.9 (10.2;11.5)	p = 0.71
**Prior MI**	2.5 (1.9;3.5)		5.8 (4.4;7.8)		10.7 (10.2;11.3)	
**No current smoking**	2.5 (1.9;3.4)	p = 0.13§	5.5 (4.2;8.7)	p = 0.001*	10.8 (10.2;11.3)	p = 0.30
**Current smoking**	2.6 (1.9;3.8)		6.7 (5.1;8.7)		10.8 (10.2;11.5)	
**Female**	2.4 (1.7;3.4)	p = 0.04*	5.7 (4.3;7.8)	p = 0.35	10.8 (10.1;11.3)	p = 0.51
**Male**	2.6 (1.9;3.6)		6.0 (4.6;7.9)		10.7 (10.2;11.4)	
**Age <65 years**	2.5 (1.9;3.4)	p = 0.67	5.9 (4.4;7.8)	p = 0.30	10.7 (10.2;11.4)	p = 0.82
**Age ≥65 years**	2.5 (1.9;3.6)		5.6 (4.2;7.7)		10.8 (10.2;11.3)	
**eGFR ≤60 mL/min**	2.5 (1.9;3.4)	p = 0.17	6.0 (4.5;8.0)	p = 0.59	10.7 (10.2;11.3)	p = 0.23
**eGFR >60 mL/min**	2.5 (1.9;3.6)		5.7 (4.3;7.8)		10.8 (10.2;11.4)	
	**Platelet count**		**Platelet distribution**		**Platelet large**	
	**10^∧^9/L**		**width, fL**		**cell ratio, %**	
**No diabetes**	227 (193;262)	p = 0.70	13.0 (12.0;14.0)	p = 0.02*	31 (26;36)	p = 0.07§
**Type 2 diabetes**	219 (187;273)		13.5 (12.0;15.0)		32 (28;38)	
**No prior MI**	223 (193;272)	p = 0.60	13.9 (12.0;15.2)	p = 0.43	33 (27;38)	p = 0.69
**Prior MI**	226 (191;263)		13.0 (12.0;14.0)		31 (27;37)	
**No current smoking**	222 (190;257)	p = 0.003*	13.0 (12.0;14.0)	p = 0.38	31 (27;36)	p = 0.40
**Current smoking**	236 (199;289)		13.0 (12.0;15.0)		32(26;38)	
**Female**	254 (221;298)	p<0.0001*	13.0 (12.0;14.0)	p = 0.45	32 (27;36)	p = 0.50
**Male**	218 (186;255)		13.0 (12.0;14.0)		31 (27;37)	
**Age <65 years**	232 (198;273)	p = 0.001	13.0 (12.0;14.0)	p = 0.28	31 (26;37)	p = 0.92
**Age ≥65 years**	220 (185;256)		13.0 (12.0;14.1)		31 (27;36)	
**eGFR ≤60 mL/min**	236 (207;279)	p = 0.0003*	13.0 (12.0;14.0)	p = 0.08	31 (26;36)	p = 0.27
**eGFR >60 mL/min**	221 (185;256)		13.0 (12.0;14.4)		32 (27;37)	

Data is presented as median (25th;75th percentile). Groups are compared using Mann-Whitney test.

p<0.05 in linear multivariate regression analyses adjusted for diabetes, prior MI, smoking, sex, age and eGFR.

p<0.10 in linear multivariate regression analyses adjusted for diabetes, prior MI, smoking, sex, age and eGFR.

MI: myocardial infarction, eGFR: estimated glomerular filtration rate.

### Clinical Characteristics, Thrombopoietin and Low-grade Inflammation

Thrombopoietin levels were significantly increased in patients with diabetes (median pg/mL [IQR]: 50 [34;67] vs. 43 [26;64], p = 0.03), smokers (51 [34;72] vs. 43 [27;62], p = 0.003) and in patients without a history of myocardial infarction (53 [41;67] vs. 43 [27;64], p = 0.03). Thrombopoietin levels did not differ between females and males (43 [26;61] vs. 45 [29;65], p = 0.40).

Hs-CRP levels were significantly augmented in smokers (median mg/L [IQR]: 1.1 [0.6;2.4] vs. 0.7 [0.4;1.5], p = 0.001) and a trend was seen in patients with diabetes (1.1 [0.4;2.3] vs. 0.8 [0.4;1.4], p = 0.06). IL-6 was significantly increased in patients aged ≥65 years (pg/mL [IQR]: 2.4 [1.5;3.0] vs. 1.9 [1.5;3.0], p<0.0001) and in patients with eGFR >60 mL/min (2.3 [1.5;3.9] vs. 1.8 [1.5;2.8], p = 0.0001).

## Discussion

To the best of our knowledge, this is the first study investigating platelet turnover parameters and the influence of low-grade inflammation and thrombopoietin in a large, homogeneous group of stable CAD patients. In our study, we found strong associations between the platelet turnover parameters IPF, IPC, MPV, PDW and P-LCR. Surprisingly, thrombopoietin was inversely related to IPF, IPC, MPV, PDW and P-LCR. Furthermore, thrombopoietin was significantly increased in patients with diabetes and in smokers. Regarding low-grade inflammation, hs-CRP correlated positively with IPC and platelet count and IL-6 correlated with thrombopoietin, but otherwise there were no associations between platelet turnover parameters and low-grade inflammatory markers.

### Platelet Turnover in Coronary Artery Disease

We found strong associations between IPF, IPC, MPV, PDW and P-LCR, which is consistent with previous studies investigating platelet turnover parameters in atherosclerotic patients [14]–[17], [20], [26], [27]. Compared with a group of healthy controls included in a previous study by our group [15], the stable CAD patients included in the present study had higher IPF, MPV and PDW, indicating increased platelet turnover in stable CAD patients. Furthermore, the presence of high IPF values (>6%) was associated with increased age, which is consistent with results from the study by Cesari et al. [14].

### Platelet Turnover and Thrombopoietin

We found an unexpected, significant, inverse relation between thrombopoietin and IPF, IPC, MPV, PDW and P-LCR. A similar inverse relation has been reported in several other studies, although in different clinical settings than ours [28]–[30]. The regulation of thrombopoietin is complex and is not fully understood. Under normal physiologic conditions, decreased platelet production and turnover rate result in increased levels of unbound thrombopoietin, thereby enabling a compensatory response of megakaryocytes to the increased demand for peripheral blood platelets [23]. One may hypothesize that the inverse relation between thrombopoietin and platelet turnover parameters found in our study could be explained by an inclination of immature platelets to take up circulating thrombopoietin, thus decreasing free thrombopoietin levels in the setting of increased platelet turnover. Others have suggested megakaryocyte mass as the major determinant of thrombopoietin levels as opposed to circulating platelet count and size [23], [29], [30]. However, we did not measure megakaryocyte mass in our study.

Limited data exits on the relation between thrombopoietin and the platelet turnover parameters IPF and IPC in stable CAD patients. In a previous study by our group, we found that thrombopoietin was an independent predictor of IPF and IPC in patients with stable CAD [20]. In a study by Seneran et al., increased levels of MPV and thrombopoietin were reported in CAD patients as compared with healthy individuals [31]. MPV and thrombopoietin were positively correlated, which is contrasting our results [31]. However, patients included in the study by Seneran et al. were in the acute phase of myocardial infarction or unstable angina as opposed to our patients with stable phase of CAD. This may offer an explanation for the contrasting results, since the interaction between thrombopoietin and thrombopoiesis may be altered during acute coronary syndrome. In accordance with this hypothesis, Lupia et al. demonstrated higher levels of thrombopoietin in patients with unstable angina than in patients having stable angina [32].

### Thrombopoietin and Clinical Characteristics

In the present study, thrombopoietin levels were significantly increased in patients with diabetes, which is in accordance with previous results from our group [20]. Furthermore, smokers had significantly augmented thrombopoietin levels. Consistent with our results, Lupia et al. demonstrated significantly increased thrombopoietin levels in smokers as compared with non-smokers [33]. However, their study was fairly small (n = 40) and the study population consisted of healthy individuals. In long-term smokers, platelet aggregability is enhanced leading to subsequent alterations in the clotting-cascade in favor of a pro-thrombotic state [34]. Platelets are capable of releasing thrombopoietin when stimulated [35]. Thus, a potential explanation of increased thrombopoietin levels in smokers could be that platelets themselves are contributors to thrombopoietin release. To the best of our knowledge, no other studies have investigated the distribution of thrombopoietin in smokers with stable CAD.

### Platelet Turnover and Low-grade Inflammation

Hs-CRP reflects chronic low-grade inflammation and is a marker of cardiovascular risk [36]. In our study, hs-CRP correlated significantly, yet weakly, with IPC, platelet count and thrombopoietin. Recently, Sahin et al. reported that hs-CRP was independently related to MPV in stable CAD patients and suggested that high MPV values may be part of the chronic low-grade inflammatory state in stable CAD patients [10]. Lupia et al. found significantly higher levels of thrombopoietin and CRP in patients with unstable angina as compared with patients having stable angina and suggested that the acute-phase response related to acute coronary syndrome may play a role in increasing thrombopoietin levels [32].

Even though thrombopoietin is considered to be the key hormone in the regulation of platelet production, it is thought to function in conjunction with numerous cytokines [23]. In vitro, IL-6 seems to act as a megakaryocyte maturation factor and infusion of IL-6 has been shown to induce modest thrombocytosis [37], [38]. Recently, in vivo experimental studies confirmed the regulatory role of IL-6 in thrombopoiesis [39], [40]. However, in the present study, including stable, high-risk CAD patients, we found no associations between IL-6 and any platelet turnover parameters, although, IL-6 and thrombopoietin were weakly associated.

### Platelet Turnover and Clinical Characteristics

In agreement with a previous study [41], we found that CAD patients with diabetes had significantly higher PDW than patients without diabetes and a trend for augmented IPC, MPV and P-LCR. A number of studies have reported significant associations between type 2 diabetes and increased platelet volume indices in CAD patients [10], [15], [42], [43]. Potential mechanisms explaining this relation include increased platelet turnover [44] and osmotic swelling of platelets on the basis of raised blood glucose and increased glucose metabolites levels [45].

Smoking is a major risk factor for thrombosis and is associated with adverse cardiovascular events, in part due to enhanced platelet aggregation [34]. We found that smokers had significantly increased IPC and platelet counts compared with non-smokers. This is consistent with previous studies reporting increased IPF in smokers compared with non-smokers [15], [46].

In the present study, patients with eGFR ≤60 mL/min had significantly higher platelet counts compared with patients having normal renal function. This is contrasting recent results by Ucar et al. who reported significantly lower platelet counts in stable CAD patients with eGFR <60 mL/min compared with eGFR ≥60 mL/min [42].

Several previous studies have reported increased MPV [7], [47] and IPF [15] in the acute phase of ST-elevation myocardial infarction. In our study investigating stable CAD patients, platelet turnover parameters did not differ between patients with or without prior myocardial infarction, suggesting the increase in platelet turnover in myocardial infarction is related to the acute event.

### Strenghts and Limitations

The overall strength of our study is the inclusion of a large, homogeneous study population with stable CAD. Our study population is very similar to the average CAD patient and thus, the present study has broad applicability. We deliberately excluded patients with low platelet counts or thrombocytosis in order to study platelet turnover in a stable population with platelet counts within the normal range. However, being an observational study, we were not able to establish causality between platelet turnover parameters, thrombopoietin and markers of low-grade inflammation. Accordingly, the conclusions from this study should be seen as being hypothesis-generating.

### Conclusion

In stable, high-risk CAD patients, platelet turnover parameters were strongly associated with each other. Thrombopoietin levels were inversely associated with platelet turnover parameters and were significantly increased in patients with diabetes and in smokers. Low-grade inflammation did not seem to have a substantial impact on platelet turnover parameters.
